# Evaluating the benefits of immunotherapy in metastatic cervical cancer: an observational retrospective analysis

**DOI:** 10.1007/s12094-025-03852-x

**Published:** 2025-02-08

**Authors:** Ana Isabel Martín-Quesada, Lina Pérez-Mendez, Natalia Dolores Pérez-Rodríguez

**Affiliations:** 1 Department of Medical Oncology, Immuno-Oncology Unit, Lausanne University Hospital, Lausanne, Switzerland; 2 Clinical Epidemiologist Department of Clinical Epidemiology and Biostatistics, Research Unit, University Hospital Nuestra Señora de Candelaria and Primary Care Management, Santa Cruz de Tenerife, Spain; 3 Networked Biomedical Research Centre (CIBER) of Respiratory Diseases, Carlos III Health Institute, Madrid, Spain; 4 Medical Oncology Department, University Hospital Nuestra Señora de Candelaria, Carretera General del Rosario 145, 38010 Santa Cruz de Tenerife, Spain

**Keywords:** Metastatic, Recurrent, Cervical Cancer, Immunotherapy, Progression-Free-Survival, Objective-Response-Rate, Real-World-Evidence

## Abstract

**Background:**

Cervical cancer is the fourth most commonly diagnosed cancer and the fourth leading cause of cancer-related death in women globally. Recent advances in immunotherapy have demonstrated promising results. This study analyses the real-world impact of adding immunotherapy for patients with stage IV cervical carcinoma.

**Material and methods:**

This longitudinal retrospective observational study included patients with stage IV cervical carcinoma in the first metastatic setting treated between 2010 and 2023 at the University Hospital Nuestra Señora de Candelaria in Tenerife, Spain. To evaluate the primary objective, patients were divided into two groups depending on whether they had received immunotherapy with pembrolizumab or not. The primary endpoint was 12-month progression-free survival in patients receiving immunotherapy compared to those not receiving it.

**Results:**

A total of 56 patients were analyzed, of whom 31 were tested for PD-L1. Among those tested, 25 patients (84%) were PD-L1 positive, and 18 of them (72%) received immunotherapy. The objective response rate was significantly higher, being 94% in the group that received immunotherapy, compared to 32% in the group that did not (*p* < 0.001). At 12 months, the cumulative probability of progression-free survival was estimated at 94.4% for the immunotherapy group versus 59.7% in the non-immunotherapy group (*p* = 0.009), with a hazard ratio of 0.116 (CI_95%_: 0015 – 0.883; *p* = 0.038). Immunotherapy alone or combined with bevacizumab showed similar progression-free survival probabilities. However, these outcomes were significantly different when compared to patients who received neither therapy (*p* < 0.001).

**Conclusions:**

Our findings confirm that immunotherapy significantly improves progression-free survival and objective response rates in metastatic cervical carcinoma, aligning with the results from the KEYNOTE-826 trial. The implementation of PD-L1 testing and the addition of immunotherapy whenever possible are challenges to be achieved in clinical practice.

## Introduction

Cervical cancer is the fourth most frequently diagnosed cancer and the fourth leading cause of cancer death in women, with an estimated 661.000 new cases and 348.000 deaths worldwide in 2022 [1].

Cervical cancer is a global public health problem, with a particularly high burden in many low- and middle-income countries. Approximately 83% of all new cervical cancer cases and 88% of all deaths occur in those countries [2,3].

Furthermore, 99.7% of cervical cancer cases are caused by persistent genital high-risk human papillomavirus infection [4,5].

Patients with persistent, recurrent or metastatic cervical cancer have historically suffered from a dismal prognosis. Short-lived responses to platinum-based chemotherapy doublets followed by rapid deterioration of quality of life and early death have been the rule, with median overall survival (OS) ranging from 7 to 12 months [6,7].

Previous studies showed that the addition of bevacizumab in combination with chemotherapy in patients with recurrent, persistent, or metastatic cervical cancer was associated with an improvement of 3.7 months in median OS [7].

Immunotherapy (IO) in cervical cancer is a promising option since programmed cell death ligand 1 (PD-L1) has a high expression in this tumour [8,9]. Therefore, the phase 2 KEYNOTE-158 trial, pembrolizumab, a Programmed Cell Death Protein 1 (PD-1) inhibitor, achieved an objective response of 14.3% in patients who had received one or more prior chemotherapy treatments for recurrent or metastatic disease and had PD-L1–positive tumors [10]. Based on this, pembrolizumab was the first immunotherapy to receive accelerated approval from the United States Food and Drug Administration in 2018 as a second-line treatment for patients with metastatic cervical cancer with positive PD-L1 expression.

Subsequently, the phase 3 KEYNOTE-826 trial explored the addition of pembrolizumab to chemotherapy with or without bevacizumab in patients with recurrent, persistent, or metastatic cervical cancer, showing a benefit in OS and progression-free survival (PFS) regardless of the expression of PD-L1, being OS 28.6 months versus 16.5 months in patients with a combined positive score (CPS) of 1 or greater [11,12].

In 2024 data was published that patients with newly diagnosed, high-risk, locally advanced cervical cancer the addition of pembrolizumab to chemo-radiotherapy treatment significantly increased PFS [13].

Currently, there are multiple clinical trials with combinations of immunotherapy, conjugated antibodies and poly-ADP-ribose polymerase inhibitors determining a new era in the treatment and prognosis of this pathology [14].

This study aims to conduct a real-world analysis of the PD-L1 assessment and the incorporation of immunotherapy into the standard regimen with platinum-taxane-based chemotherapy plus or minus bevacizumab in PFS at 12 months. Preliminary data were presented at the European Congress on Gynaecological Oncology, now extending the findings in a comprehensive way [15].

### Methods

This observational study employed a real-world design involving female patients with de novo or recurrent stage IV cervical carcinoma, diagnosed through biopsy and computed tomography (CT) in the first-line metastatic setting. Retrospective data were collected between 2010 and 2023 at the University Hospital Nuestra Señora de Candelaria in Tenerife (HUNSC), Spain.

In our centre, PD-L1 testing began in early 2021. To evaluate the primary objective, patients were divided into two groups depending on whether they had received immunotherapy or not (IO + group and IO- group). The IO + group comprised patients who had obtained a PD-L1 result by combined positive score (CPS) ≥ 1 and received immunotherapy with pembrolizumab. The IO-group consisted of the sum of patients treated before PD-L1 testing was available, those treated after the implementation of PD-L1 testing but with a negative result (CPS less than 1) and those with a positive result but who were not candidates for immunotherapy due to comorbidities or clinical situation.

We followed the Strengthening the Reporting of Observational Studies in Epidemiology (STROBE) guidelines [16]. This study was approved by the Ethics Committee of the Canary Health Service.

### Assessments

1. PD-L1: PD-L1 expression was assessed in both tumour and inflammatory cells. The CPS was calculated as the number of PD-L1-positive cells (tumour cells, lymphocytes and macrophages) divided by the total number of viable tumour cells, multiplied by 100 (17). PD-L1 was considered positive with a CPS ≥ 1. We use the 22C3 clone from Dako Commercial.
2. Imaging and Response Evaluation: Computed tomography (CT), magnetic resonance imaging (MRI), and the Response Evaluation Criteria in Solid Tumours 1.1 (RECIST 1.1.) (18) were used to assess complete response (CR), partial response (PR), stable disease (SD) and disease progression (PD) during scheduled follow-up at 12 months.

### Variables

The primary independent variable in the study was the presence or absence of immunotherapy. The main dependent variable was the disease progression assessed 12 months after the start of treatment. Secondary variables included age at diagnosis, histology, performance status by the Eastern Cooperative Oncology Group (ECOG) scale [19] and previous treatments received in the local or locally advanced setting such as surgery and radiotherapy and current treatment with chemotherapy and bevacizumab for the metastatic disease.

### Endpoints

The primary endpoint was to determine the cumulative PFS at 12 months in the IO + group versus the IO- group, the hazard ratio (HR) and incidence risk (IR) calculated at 12 months. The IR was defined as the number of patients who progressed within 12 months after starting first-line metastatic treatment, per 1000 patients at risk.

The secondary objectives included the comparison of the objective response rate (ORR) between the IO + and IO- groups and the cumulative PFS at 12 months in patients treated with or without bevacizumab and in addition or not to immunotherapy.

Although for descriptive purposes, an analysis of the available follow-up period was performed for each patient included (median and percentiles), we considered the PFS at 12 months as the indicator that better oriented the medical oncologist.

### Study population

Patients diagnosed via biopsy and CT scan with metastatic cervical carcinoma either de novo or recurrent and followed by the medical oncology service at the HUSNC. The patients were selected from the existing registry of the hospital's monographic gynaecological tumour clinic.

### Sample size

All patients with PD-L1 determination after the beginning of 2021 until 2023 were included. For the pre-2021 period, patients were selected consecutively in reverse chronological order until a sufficient sample size was reached.

### Inclusion criteria

- A complete clinical history available in our hospital's electronic system, allowing for the collection of clinical, histological, molecular and radiological data.

### Exclusion criteria

- Incomplete clinical history, particularly for cases from before the establishment of the PD-L1 test, where key information was missing.

### Statistical analysis

Data are summarised as frequencies for categorical variables, mean ± standard deviation for normally distributed variables and median (25–95th percentile) for non-normally distributed quantitative variables. Comparisons between groups were performed using Pearson’s Chi-squared test, one-way ANOVA, the Kruskal–Wallis H-statistic, the Mann–Whitney U- rank test and Student t-test. Kaplan–Meier analysis was used for comparison of the cumulative PFS between the two groups previously described. The statistical significance was evaluated using the log-rank test and Hazard Ratio by Cox Regression analysis were calculated.

A statistical analysis of β-power between the two groups was performed at the end of the study due to the reduced sample obtained in "IO + group" (by the few patients with cervical carcinoma de novo or relapsing stage IV, between 2021–2023.)

Bar chart, box-plots and survival graphics were plotted. Significance level was established as a *p* < 0.05. Calculations were made using the Statistical Package for the Social Sciences (SPSS) 26.0 (Chicago, Illinois, United States of America).

## Results

A total of 56 female patients were included in the study. Out of the total patient cohort, 31 were diagnosed after 2021, with 25 (84%) testing positive for PD-L1 and 18 (72%) receiving immunotherapy, defining the IO + group. The remaining 38 patients, which included those treated before PD-L1 testing was available (*n* = 25), those who tested negative (*n* = 6) and those tested positive for PDL1 but due to comorbidities or late diagnoses were not candidates for immunotherapy (*n* = 7), were categorised as the IO- group (*n* = 38).

The average age was 53.5 ± 12, with no significant difference between the IO + group and the IO- group. The most common histological subtype was squamous cell carcinoma (82.1%), again with no significant differences between groups. However, the distribution of patients' ECOG performance status scores differed significantly between the two groups (*p* = 0.014).

Regarding previous treatments received, there were no differences between the groups in the percentage of patients who underwent surgery (25.0%) or radiation therapy (76.8%) in the past.

For the first-line metastatic setting all the patients were treated with platinum and paclitaxel every 3 weeks. Additionally, bevacizumab was used in a lower proportion of patients in the IO- group (*p* = 0.001). This fact was adjusted in the combined treatment analysis.

The comparative analysis of covariates is summarised in Table 1*.*

**Table 1:** Baseline Characteristics of Patients

	All n = 56	IO-Negative group n = 38	IO-Positive group n = 18	*p*-value
Variables, units				
Age (mean ± SD)	54 ± 12	53 ± 12	53 ± 13	0.734
Grade, n (%)				0.747
1	3 (5.4%)	2 (5.3%)	1 (5.6%)	
2	18 (32.1%)	11 (28.9%)	7 (38.9%)	
3	35 (62.5%)	26 (65.8%)	10 (55.6%)	
Histology, n (%)				0.217
Squamous	46 (82.1%)	28 (73.7%)	18 (100%)	
Adenocarcinoma	6 (10.7%)	6 (73.7%)	0 –	
Adenosquamous	2 (3.6%)	2 (53%)	0 –	
Clear cell	1 (1.8%)	1 (2.6%)	0 –	
Carcinosarcoma	1 (1.8%)	1 (2.6%)	0 –	
ECOG, n (%)				0.014
0	21 (37.5%)	9 (23.7%)	12 (66.7%)	
1	22 (39.3%)	19 (50.0%)	3 (16.7%)	
2	11 (19.6%)	8 (21.1%)	3 (16.7%)	
3	2 (3.6%)	2 (5.3%)	0	
Surgery, n (%)				0.099
No	42 (75.0%)	31 (81.6%)	11 (61.1%)	
Yes	14 (25%)	7 (18.4%)	7 (38.9%)	
Radiotherapy, n (%)				0.638
No	13 (23.2%)	10 (26.3%)	3 (16.7%)	
Yes	43 (76.8%)	28 (73.7%)	15 (83.3%)	
Bevacizumab, n (%)				0.001
No	30 (53.6%)	26 (68.4%)	4 (22.2%)	
Yes	26 (46.4%)	12 (31.6%)	14 (77.8%)	
Follow-up (months)				0.001
Median [P_25_—P_75_]	7 [3–13]	6[2–12]	8.5 [6.7–20.5]	
Min -Max	1–84	1–84	3–26	

IO = Immunotherapy; SD = Standard Deviation;ECOG = Eastern Cooperative Oncology Group; n = number; P_25_ = 25th percentile; P_75_ = 75th percentile

### Differences in ORR

ORR in IO + group was 94.5%, versus ORR IO- group was 31.6% (*p* < 0.001). CR, PR, SD or PD for each treatment group are shown in Table 2*.*

**Table 2:** Comparison of responses between the group with immunotherapy with pembrolizumab versus the group without immunotherapy

	All *n* = 56	“IO Negative group” *n* = 38	“IO Positive group” *p*-value *n* = 18	*p* < 0.001
RECIST 1.1 n (%)				
Complete Response	13 (23.2%)	6 (15.8%)	7 (38.9%)	
Partial Response	16 (28.6%)	6 (15.8%)	10 (55.6%)	
Stable Disease	11 (19.6%)	11 (28.9%)	0	–
Progressive Disease	16 (28.6%)	15 (39.5%)	1 (3.6%)	

### Differences in PFS at 12 months

The descriptive analysis showed a difference in the follow-up times between the IO- group: minimum 1 months-maximum 84, median 6 months (2–12) and the IO + group: minimum 3 months-maximum 26, median 8.5 months (6.7–20.5) (Table 1). Therefore, for the comparative analysis of cumulative PFS, we decided to focus on the first 12 months post-treatment, as outlined in the methods section.

**Fig. 1:**
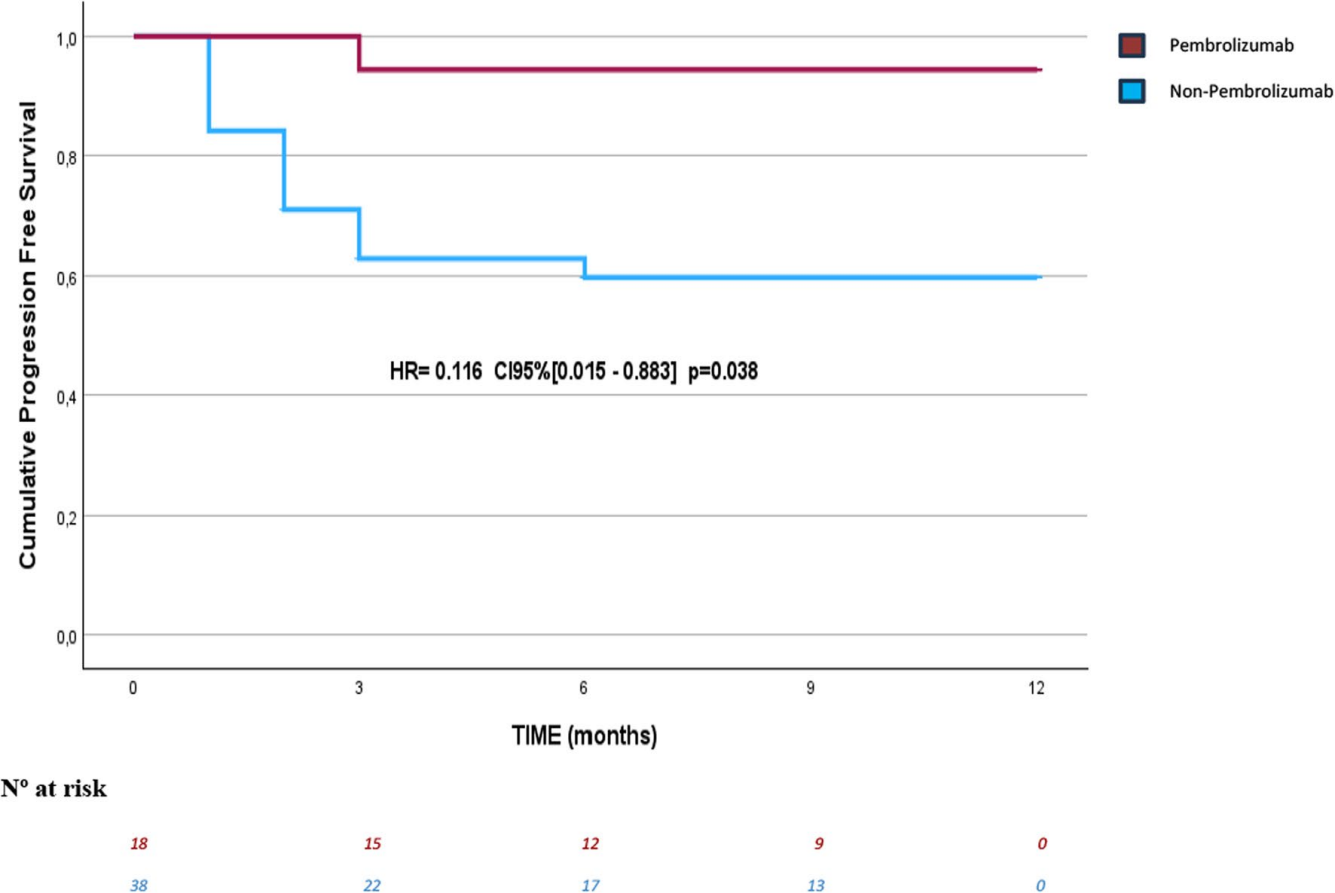
Progression-Free Survival at 12 months with Pembrolizumab versus Non-Pembrolizumab. *HR* hazard ratio, *CI* confidence interval

The 12-month cumulative PFS assessed by Kaplan–Meier demonstrates a significant difference between the groups (*p* = 0.009), with the IO + group at 94.4% compared to 59.7% in the IO- group (Fig. 1).

The crude HR of relative risk at 12 months was 0.116 (CI_95%_: 0.015–0.883; *p* = 0.038) and the incidence rate in IO + group patients was 6.3 per 1000-patients at risk, while for IO- group patients was 62.2 per 1000-patients at risk (Table 3).

**Table 3:** Comparison of incidence rates between the group with immunotherapy with pembrolizumab versus the group without immunotherapy

	“IO positive group”	“IO negative group”	
			*p* = 0.038
Hazard Ratio	0.116	95.0% CI (0.015–0.883)	
Incidence Rate	6.3 per 1000-patients at risk	62.2 per 1000-patients at risk	

CI = Confidence Interval; IO = Immunotherapy

### Addition of bevacizumab

**Fig. 2:**
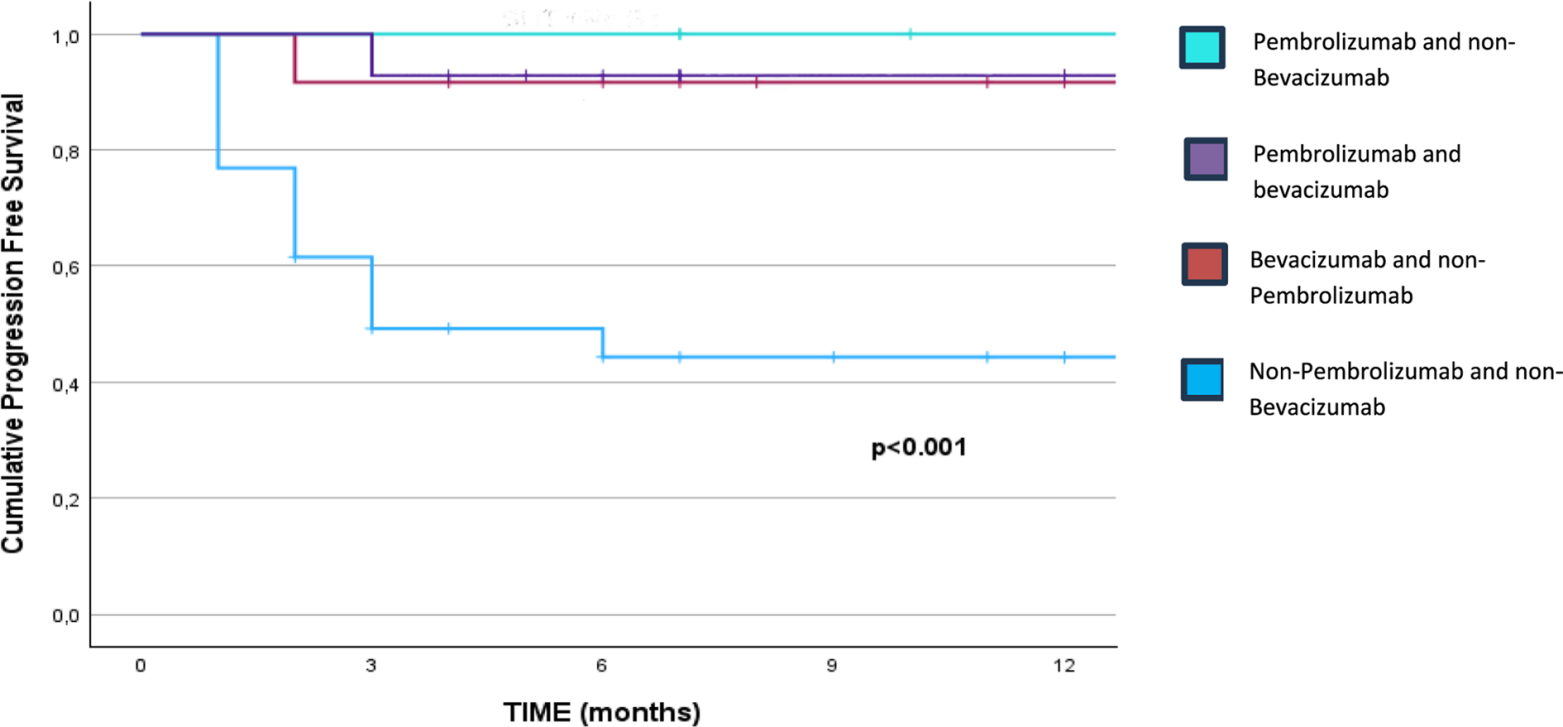
Cumulative Progression-Free Survival at 12 months comparing Pembrolizumab versus Non-Pembrolizumab and Bevacizumab versus Non-Bevacizumab

The impact of adding bevacizumab on PFS is shown in Fig. 2. IO alone or combined with bevacizumab maintained similar probabilities of PFS at 12 months, all above 90%, and significantly different when compared to patients who did not receive either therapy (12-month-PFS was 44%, *p* < 0.001).

### β-power analysis

At the end of the study, the IO + and IO- groups showed cumulative PFS of 94% and 40%, respectively (Fig. 1). The statistical power of this study to detect a difference as observed, with a significance level of 0.05 (95% confidence level), was greater than 80% (calculated using the OPeEpi tool: https://www.openepi.com/Power/).

## Discussion

### Summary of main results

This study shows the evolution of the management of metastatic cervical carcinoma over the last decade in our centre. We analyzed the group of patients who either received or did not receive pembrolizumab along with standard chemotherapy and, in some cases, bevacizumab. Demographic and disease characteristics were generally well balanced but with significant differences in ECOG performance status. All patients received platinum-taxane-based chemotherapy for the first metastatic setting but there were differences in receiving or not bevacizumab, having received it in a higher proportion in the group that also underwent immunotherapy (77.8% versus 31.6%, *p* = 0.013). In terms of outcomes, we observed a benefit in both 12-month PFS and ORR for the group that received immunotherapy, with CR and PR response rates equally higher than the non-pembrolizumab group (CR rates of 38.9% versus 15.8% and PR rates 55.6% versus 15.8%, *p* < 0.001).

### Results in the context of published literature

Following the publication of the results of the KEYNOTE-826 study demonstrating the benefit of adding pembrolizumab for patients with recurrent, persistent, or metastatic cervical cancer, the management and prognosis of these patients has changed dramatically (11,12). In this study, the addition of pembrolizumab to treatment with chemotherapy plus-minus bevacizumab versus chemotherapy plus-minus bevacizumab resulted in benefits in PFS, OS and ORR.

In our series, the benefit in 12-month PFS is evident, with statistically significant differences observed. Notably, the cumulative 12-month PFS in our series was higher than that reported in the KEYNOTE-826 trial, with a PFS of 94.4% for the IO + group compared to 59.7% for the IO- group. In contrast, the KEYNOTE-826 trial reported a PFS of 45.5% for the CPS > 1 population receiving immunotherapy versus 34.1% for those who did not (95% CI). In addition, our HR for the 12-month PFS was 0.116, which is wide (0.015–0.883) including the HR and the confidence interval of the HR for the 12-month PFS of the KEYNOTE-826, so the results seem to be in accordance. At present, the median PFS has not yet been reached; however, based on current results, it is expected to exceed 12 months in both groups, suggesting outcomes at least similar to or even better than those in the KEYNOTE-826 trial.

Another key objective was to evaluate the ORR. In our series, the ORR was 94.5% in the IO + group, compared to 31.6% in the IO- group (*p* < 0.001). By comparison, in the KEYNOTE-826 trial, patients with CPS > 1 had an ORR of 68.1% in the immunotherapy group versus 50.2% in the group without. The CPS > 1 cutoff is used for these comparisons, as this matches the population in our analysis that received immunotherapy. Nevertheless, various factors such as the difference in sample size and different performance status may be relevant to these results.

A further major concern today is the uncertainty regarding the addition of bevacizumab to immunotherapy treatment. Past research indicated that including bevacizumab in the chemotherapy regimen resulted in a median OS increase of 3.7 months, as mentioned before (7).

In the KEYNOTE-826 study in the intention to treat population the OS in patients who did or not receive bevacizumab presented a HR of 0.63 (95%CI, 0.47–0.87) versus 0.74 (95%CI, 0.53–1.04) respectively. Regarding the PFS, the HR for the bevacizumab group was 0.61 (95%CI, 0.47–0.79) versus 0.74 (95% CI, 0.54–1.01) (11,12,20). These results suggest that not including bevacizumab might slightly increase the risk of death or progression.

In our series, immunotherapy, whether administered alone or in combination with bevacizumab, resulted in similar 12-month PFS rates, with a PFS superior to 90% in all the cases (*p* < 0.001). These outcomes, nevertheless, were significantly different when compared to patients who received neither therapy, with a PFS of 44% (*p* < 0.001). However, the small number of patients who received bevacizumab must be taken into account.

Furthermore, based on the results published it has been attempted to standardise the performance of PD-L1 in all patients in this setting. The results show that in our centre the determination of PD-L1 is now performed in all patients with stage IV cervical carcinoma. In patients in whom it had been determined, 84% had a CPS ≥ 1, which is consistent with the KEYNOTE-826 results (89% of patients with CPS ≥ 1).

Notably, in the IO + group the ECOG performance status varied compared to the IO- group. This could be due to the fact that patients now consult earlier before they develop more advanced symptoms of the disease.

### Strengths and weaknesses

The strengths of this analysis include a comprehensive analysis of a decade of data from our centre, providing a longitudinal perspective on the management of this disease. The study highlights the real-world application and outcomes of immunotherapy. However, the study's weaknesses include its retrospective nature and the potential for information bias due to data collection from medical records. Additionally, the study was conducted in a single centre with a limited sample size, which may not fully represent broader patient populations.

### Implications for practice and future research

The results of this study suggest that the implementation of immunotherapy in the treatment of metastatic cervical carcinoma can lead to significant improvements in PFS, although these improvements may vary based on patient characteristics and selection. The findings highlight the need for standardised procedures for determining PD-L1 as well as the importance of early consultation. The study highlights the changing panorama of metastatic cervical carcinoma treatment, emphasizing the need for ongoing evaluation and adaptation of clinical practices.

## Conclusions

Our experience shows that immunotherapy significantly improves PFS and ORR in patients with metastatic cervical carcinoma, supporting the KEYNOTE-826 trial findings. These results highlight the importance of integrating immunotherapy into treatment plans and the need for standardised PD-L1 testing. Further research, including larger multi-centre studies, is necessary to optimise treatment strategies and confirm these findings. These conclusions underscore the evolving landscape of metastatic cervical carcinoma treatment and recent opportunities for these patients.

## Data Availability

All data are available upon request.
